# A *TRPV1* common missense variant affected the prognosis of ischemic cardiomyopathy

**DOI:** 10.1097/MD.0000000000029892

**Published:** 2022-07-29

**Authors:** Shiyang Li, Lei Xiao, Yang Sun, Senlin Hu, Dong Hu

**Affiliations:** a Division of Cardiology, Panzhihua Central Hospital, Panzhihua, China; b Division of Cardiology, Department of Internal Medicine, Tongji Hospital, Tongji Medical College, Huazhong University of Science and Technology, Wuhan, China; c Hubei Key Laboratory of Genetics and Molecular Mechanisms of Cardiological Disorders, Huazhong University of Science and Technology, Hubei Province, Wuhan, China.

**Keywords:** heart failure, ischemic cardiomyopathy, prognosis, TRP, whole exome sequencing

## Abstract

The purpose was to identify the Transient receptor potential (TRP) superfamily gene variants associate with the prognosis of ischemic cardiomyopathy (ICM). A whole-exome sequencing study involving 252 ICM and 252 healthy controls participants enrolled from March 2003 to November 2017. Optimal sequence kernel association test and Cox regression dominant was conducted to identify the cause genes of TRP with ICM and association of common SNPs with prognosis of ICM. Rs224534 was verified in the replication population. Besides, the expression of TRPV1 was detectable in human failed heart ventricular tissues. The TRPs was not associated with the risk of ICM (*P* > .05). Rs224534 was significantly associated with the prognosis of ICM (Hazard ratio, 2.27, 95%CI: 1.31–3.94; *P* = 3.7 × 10^–3^), in the replication cohort, (hazard ratio 1.47, 95%CI: 1.04–2.07; *P* = 2.9 × 10^–2^), and in combined cohort hazard ratio 1.62 (95%CI: 1.21–2.18; *P* = 1.1 × 10^–3^). The common SNP of TRPV1 (rs224534) is associated with the prognosis of ICM, and homozygote rs224534-AA showed an unfavorable prognosis of ICM in the dominant model tested. Genotyping the variant may benefit to further progress judgment of ICM.

## 1. Introduction

Coronary artery disease (CAD) and ischemic cardiomyopathy (ICM) are the prevailing compounds of HF^[1]^ and many patients who suffer shortly life expectancy as the damaged myocardium affects systolic and diastolic.^[2]^ ICM remains a progressive disease, although classical pharmacotherapy, revascularization has made great improvement for patient’s quality life and outcomes.^[3]^ Meanwhile, genetic factors involved in the pathological process of ICM and might have relevant to the severe prognosis for various etiology of HF,^[4]^ while, new variants of individuals impart the effect of overall genetic risk which needs further exploration and understanding.

Calcium ions (Ca^2+^) are considered to play an irreplaceable role in cell physiopathology and cell signaling which as the second messengers regulate myocardial contraction in cellular responses, also involved in heart failure.^[5]^ The transient receptor potential cation (*TRP*) channels, as nonselective cation channels with a variable degree of Ca^2 +^ permeability, are involved in the development of the cardiovascular system.^[6]^ A total of 6 members namely *TRPC, TRPV, TRPM, TRPA, TRPP*, and *TRPML*^[7]^ harboring 28 genes was identified to form the human *TRP* superfamily because of sharing the same transmembrane structure. *TRP* channels are not typical voltage receptor, can be activated by oxidative stress,^[8]^ mechanical stress,^[9]^ cellular environment,^[10]^ inflammatory factor,^[11]^ and crosstalk with the downstream signal path to affect cell function by regulating intracellular ca^2 +^ permeation. The function of *TRP* is associated with cardiovascular diseases including heart failure,^[12]^dilated cardiomyopathy,^[13]^atherosclerosis,^[14]^ ischemic cardiomyopathy.^[15]^ Variations in proteins that code TRP superfamily gene may lead to an effect of pathogenic states of cardiovascular diseases.^[16,17]^ However, the association between Variations of TRP and ICM is not understood. We assumed that the gene polymorphism of the *TRP* channel may influence the prognosis of ICM. Here, we investigate the hypothesis by whole-exome sequencing-which enrolled ICM cases with significantly enlarged left ventricle to profile genomic variant results of TRP and inspect to link with the clinical prognosis of HF, which could be replicated in another cohort and would be useful for novel biomarker generation, related to the prognosis of ICM.

## 2. Methods

### 2.1. Discovery population

The study followed the principles of the declaration of Helsinki and approved by the Institutional Ethics Committee of Tongji Hospital. A total of 252 ICM patients were Chinese Han, up recruited gave written informed consents. Meanwhile, 252 healthy controls were collected from communities of Wuhan. ICM was diagnosed based on coronary heart disease: presence of previous myocardial infarction, ≥75% stenosis in 1 or more major epicardial coronary arteries or presence of previous percutaneous coronary intervention, coronary artery bypass grafting; left ventricular ejection fraction < 50%; left ventricular end-diameter (LVEDD) > 117% of the predicted value corrected for age and body surface area in significant coronary artery disease.^[18]^ Besides, patients with well-controlled hypertension and diabetes were also included and continuously enrolled from hospitalized patients in Tongji Hospital. The enrolled time is from March 2003 to November 2017. The primary endpoint was defined as heart transplantation or cardiovascular death. The clinical characteristics of individuals were summarized in Table 1. The risk factors were as follows: gender, age, hypertension, hyperlipemia, diabetes mellitus, current smoking. Meanwhile, β-blocker take as an adjusted factor was collected at different time points. The rate of follow-up compliance was 100% (n = 252). The average age was 65 years (65.3 ± 9.3). The male component is 85.7%. Details of the study cohort regarding inclusion criteria, data collection, and definition of risk factors were offered in the online Data Supplement (Supplemental Digital Content, http://links.lww.com/MD/G903).

**Table 1:** Baseline characteristics of the study sample.

Cohort	Discovery population (n = 252)	Replication population (n = 1042)
Male, n	216 (85.71%)	686 (65.80%)
Mean age (yr)	65.26 ± 9.31	64.22 ± 11.64
Hypertension (%)	158 (62.54%)	970 (90.31%)
Diabetes (%)	88 (34.90%)	402 (38.57%)
Current/ex-smoker (%)	121 (48.00%)	402 (38.96%)
Cerebrovascular disease	22 (8.73%)	141 (13.53%)
Carotid atherosclerosis	14 (5.38%)	/
Acute coronary syndrome	189 (75.00%)	122 (11.67%)
Myocardial infarction	129 (51.32%)	72 (6.91%)
Unstable angina pectoris	57 (23.02%)	50 (4.77%)
Atrial fibrillation	20 (8.00%)	8 (0.78%)
Ventricular arrhythmia	25 (10.00%)	/
Coronary artery bypass surgery	7 (2.78%)	3 (0.29%)
Ventricular aneurysm	12 (4.67%)	/
Percutaneous transluminal coronary intervention	149 (59.21%)	43 (4.09%)
NYHA		
II	97 (38.67%)	560 (53.74%)
III	99 (39.28%)	291 (27.93%)
IV	42 (16.66%)	177 (16.99%)
Blood pressure (mm Hg)		
Systolic	130.91 ± 22.79	132.60 ± 23.44
Diastolic	80.41 ± 14.32	79.07 ± 13.87
Heart rate (bpm)	82.07 ± 18.35	79.04 ± 17.20
TC (mmol/L)	3.71 ± 0.98	3.97 ± 2.32
TG (mmol/L)(median)	1.27 ± 0.83	1.46 ± 1.15
HDL (mmol/L)	0.91 ± 1.34	1.03 ± 0.60
LDL (mmol/L)	2.32 ± 0.89	2.33 ± 0.97
ALT(U/L)	28.09 ± 22.00	42.84 ± 193.55
AST(U/L)	37.91 ± 44.85	55.63 ± 246.04
Glu (mmol/L)	6.61 ± 2.41	7.11 ± 4.12
Cr (mmol/L)	91.60 ± 96.51	111.01 ± 157.56
NT-proBNP (pg/mL)	3948.5 (1761.5,7592.0)	1025 (195.5,3395)
LVEF (%)	34.17 ± 11.15	51.88 ± 14.64
LVID (mm)	64.28 ± 6.90	52.36 ± 9.04
LVAD (mm)	44.76 ± 8.26	38.69 ± 6.68
LVSD (mm)	9.67 ± 1.64	10.10 ± 1.75
LVPW (mm)	9.80 ± 1.74	9.81 ± 1.30
Beta-blocker (%)	0.43	0.56
Digosin (%)	0.24	0.24
Diuretic (%)	0.48	47.10
Aldosterone antagonist (%)	39.25	39.82
ACEI (%)	46.54	52.85

### 2.2. Replication population

The replication cohort objectives were recruited in Tongji Hospital from January 2009 to October 2014. Patients were enrolled with the same heart failure criteria as the discovery population. Replication cases were coming from our previously study.^[19]^ A total of 1042 patients had a diagnosis of coronary heart disease and accomplished the follow-up, 12 failed genotyping, and 12 lacked information, a total of 1018 patients were successfully enrolled at last. The average age was 64 years (64.2 ± 11.6) and 34.2% was female. The primary endpoint was defined as heart transplantation and/or cardiovascular death. The spectrums of etiology and clinical characteristics were demonstrated in Table 1. Details were offered in method of supplemental materials (Supplemental Digital Content, http://links.lww.com/MD/G903). The baseline of 252 controls was offered in Supplemental Table 4 (Supplemental Digital Content, http://links.lww.com/MD/G907). The study was approved by the Institutional Ethics Committee of Tongji Hospital. All patients gave written informed consent before enrolling.

### 2.3. Human myocardial samples

Myocardial samples of 8 ICM patients were used in this study which was approved by the Institutional Ethics Committee of Tongji Hospital (Wuhan, China). In all specimens, the ventricular free wall below the papillary muscle was frozen in liquid nitrogen then kept at -80°C until use. Clinical characteristics of the study samples contained contractile parameters of echocardiography were summarized in Supplemental Table 5 (Supplemental Digital Content, http://links.lww.com/MD/G908).

### 2.4. Whole exome sequencing of discovery population

Tiangen commercially available kits (Tiangen, Beijing, China) were used to extract genomic DNA from peripheral blood leukocytes. Illumina Hiseq X ten sequencer was used to sequence the amplicons. Insertion and deletion were detected by GATK version 3.4. ANNOVAR was used to annotate variants. Raw sequence reads were aligned to the hg19 human reference genome (GRCh37 Genome Browser) using Burrows-Wheeler Alignment Tool (BWA). Removing duplicated reads was used with Picard (http://picard.sourceforge.net). Over 95% of the read coverage of the target region was ≥ 20x. PLINK and Genetic principal components analysis (PCA) was used for imputation, Hardy-Weinberg equilibrium, and cohort structure. VCF tools were used to extract variants of the TRP superfamily from the WES data pool.

### 2.5. Gene, SNP selection, and workflow

**Figure 1.**
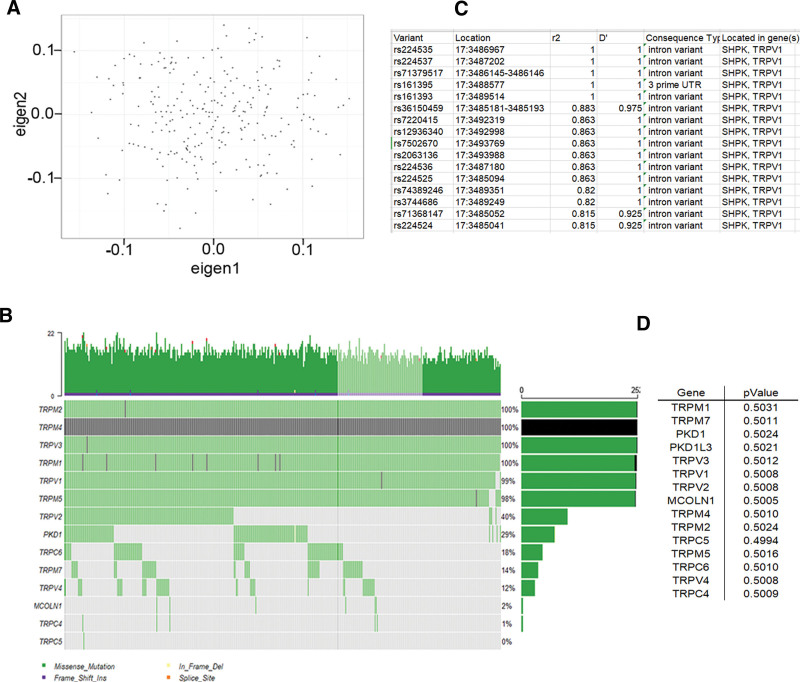
TRP has not association with the risk of ICM. (**A**) The workflow of the study. The principal component analysis (PCA) of whole-exome sequencing was performed to populations’ construction defined by smart PCA, the result demonstrated the consistency of study (**C**). Each column represents 1 of the individuals, and each row represents a gene.The genomic landscape of TRP and mutational signatures. The waterfall plot shows the top 14 most commonly mutated genes of TRP in patients with ICM. The bar chart on the left is the gene’s name, and the right is mutation frequency. There is not association between TRP and risk of ICM which listed in D, and left is the gene’s name; right is *P*-value by using the SKAT-O test compared from 252 cases, and 252 controls (**D**).

TRP superfamily consisting of 28 genes from 6 subsets may involve in cardiovascular disease. The variants of those genes were acquired from the whole-exome sequencing of the Discovery population; hence, the focus of the study concentrated on the CDS region of target genes. The workflow was offered in Figure 1A. Firstly, an optimal sequence kernel association test was performed to explore the cause genes of TRP of ICM. Secondly, TagSNPs were selected at MAF ≥ 0.05. SNPs in high linkage disequilibrium with the variants were identified using HaploReg v4.1 (https://pubs.broadinstitute.org/mammals/haploreg/haploreg.php). Besides, SNPs associated with outcomes of ICM tested with the Cox proportional hazards dominant model adjusted for gender, age, hypertension, hyperlipemia, diabetes mellitus, current smoking, and β-blocker treatment. And then, the target site was validated in a repetition cohort holding with 1018 cases and performed combine analysis in both cohorts.

### 2.6. SNP genotyping

The genotype probe of rs224534 came from ABI (USA, 4351379). Using TaqMan 5’-nuclease assay on the TaqMan 7900HT Sequence Detection System (Applied Biosystems, Foster City, California) was under the following conditions: 10 minutes at 95°C (enzyme activation) followed by 40 cycles at 95°C for 15 seconds and 60°C for 1 minute (annealing/extension). Procedure of the genotyping was consistent with our previous report.^[19]^ The genotype of failed heart was confirmed by Sanger sequencing with the primers listed in Table S1, http://links.lww.com/MD/G904.

### 2.7. Plasmid construction, cell culture, and transient transfection

Expression plasmids of rs224534, the coding region of TRPV1 gene were constructed with pcDNA3.1 through amplification of human cDNA derived from the above-mentioned human heart tissue. Related primers (including mutagenic primers) were offered in Supplemental Table S2 (Supplemental Digital Content, http://links.lww.com/MD/G905). Fast Mutagenesis System (Beijing TransGen Biotech Co., Ltd.) was performed to construct of point mutation. HEK293T came from American Type Culture Collection (ATCC), and cultured in complete DMEM medium with 10% fetal bovine serum (FBS; Gibco) under standard conditions. Lipofectamine™ 2000 (Invitrogen) was used to transfect empty vector pcDNA3.1 or to construct plasmids of rs224534. Sequences of primers for plasmids construction offered in Table S2, http://links.lww.com/MD/G905.

### 2.8. Western blot analysis

Homogenate of Myocardia of left ventricles or lysate of the 293T cell after transfected 48h was centrifuged at 12,000g for 20 minutes at 4°C. Supernatants were collected to determine concentrations by bicinchoninic acid (BCA) colorimetric assay. Cell lysates were then resolved by 10% SDS-polyacrylamide gel electrophoresis and transferred to polyvinylidene difluoride (PVDF) membranes. After blocking with 5% nonfat milk, blots were probed with TRPV1 (Abcam, UK, ab3487) and GAPDH antibody (Santa Cruz Biotechnology, USA, sc-293335). Bands were visualized by enhanced chemiluminescence reagents (Pierce Chemical, Rockford, IL) and quantified by densitometry.

### 2.9. Quantitative real-time PCR

Using TRIzol reagent kit (Invitrogen, Carlsbad, CA) isolated total RNA from in peripheral blood lymphocytes of 153 coronary artery disease (CAD) cases (baseline offered in Supplemental Table 4, Supplemental Digital Content, http://links.lww.com/MD/G907) failing heart tissue and lysate of the transfected 293T cell, and then EasyScript First-Strand cDNA Synthesis SuperMix (TransGen Biotech, Beijing, China) used to reverse-transcribed 250 to 500 ng RNA. We used custom-designed SYBR Green (Applied Biosystems Inc.) to perform QPCR analyses for human *TRPV1* and internal reference gene (GAPDH) transcripts. To avoid experimental error, triplicate replicated had to be done for all experiments. Related primer sequences were provided in Supplemental Table 3 (Supplemental Digital Content, http://links.lww.com/MD/G906).

### 2.10. Statistical analysis

Baseline characteristics of 2 cohorts including original disease, parameters of Echocardiography, and laboratory index were evaluated with descriptive statistics. Continuous data are expressed as mean ± SEM. Categorical variables are exhibited by frequency and percentage. The optimal sequence kernel association test (SKAT-O) was used to evaluate the association between genes of TRPs and ICM risk. Cox proportional hazards dominant model was performed to analyze the association of SNPs and prognosis of HF with adjusted or unadjusted the traditional risk factors. *P* < .05 was considered to be significant. Linkage disequilibrium (LD) and polymorphisms were tested with Haploview version 4.1 and Hardy-Weinberg equilibrium using χ^2^. Data analyses were performed using SPSS 24.0 (SPSS, Inc., Chicago, IL) for Windows (Microsoft Corp, Redmond, WA).

## 3. Results

### 3.1. Characteristics of the objectives

From March 2003 to June 2017, a total of 252 cases in discovery cohort have coronary heart disease, which affirmed by typical myocardial infarction graph of electrocardiogram, percutaneous coronary angiography, previous history of coronary artery bypass surgery and previous myocardial infarction; with cardiac enlargement and cardiac dysfunction. Compared to the replication cohort, the discovery cases have more gravely of CHD whose half suffering incidents of myocardial infarction, nearly 60% accomplished PCI, and 75% experienced ACS. Majority participators of discovery group have significantly enlarged left ventricle (LVID 64.28 ± 6.90 mm) and came down significantly of heart function (LVEF 34.17 ± 11.15%). The median of NT-proBNP, LVAD, LVSD, LVPW was 3948.5 pg/ml, 44.8 mm, 9.7 mm, 9.8 mm, respectively. The baseline characteristics of the discovery and replication cohort are displayed in Table 1.

### 3.2. Whole-exome sequencing association analysis

**Figure 2.**
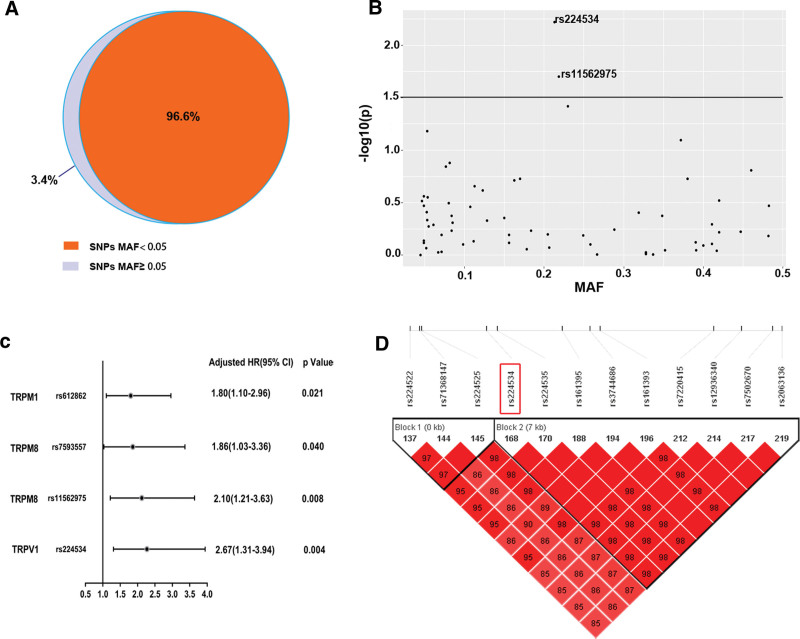
Rs224534 is associated with the prognosis of heart failure in the discovery cohort. (A) Number of target SNPs (MAF > 0.05) found in holistic variants with WES of TRP superfamily. (B) scatter diagram indicators *P*-value and MAF for common variants of TRP superfamily genes associated with outcomes of heart failure. Rs224534 is the most statistically significant SNP with cox analysis of dominant model vs others. (C) Forest map illuminates significant SNPs with HF in discovery cohort. (D) Linkage map displays rs224534 strong linkage disequilibrium sites of TRPV1.

A total of 252 ICM cases of leukocyte-derived DNA was executed WES, the sequencing depth ≥ 20X. Output variants were processed by extensive quality control, unfolded 1868 variants in exon regions of TRP superfamily, 2 indels, and 998 single-nucleotide variants. PCA (Fig. 1B) confirmed the heteroplasmy levels and genetic relationships. Variants Harbored in TRP Genes of ICM were shown in Figure 1C, and they were not associated with the risk of ICM by the SKAT-O test in Figure 1D (*P* > .05). The current studying is to focus on the association between common SNPs in the CDS region and prognosis of ICM; hence, MAF ≥ 0.05 was selected as the threshold of candidate genes based on our study and the 1000 Genomes website of Chinese Han (http://www.internationalgenome.org/1000-genomes-browsers). After filtering the deleterious variants (damaging missense, splicing site, nonsense, and frameshift indel variants), a total of 64 SNPs is a common variant with MAF ≥ 0.05 as the highlight targets (Fig. 2A). By Haploview 4.0, 5 SNPs were in strong LD with rs254434 (D’ = 1, r^2^ = 1) (Fig. 2D).

### 3.3. Deduced target SNPs in the discovery cohort

**Figure 3.**
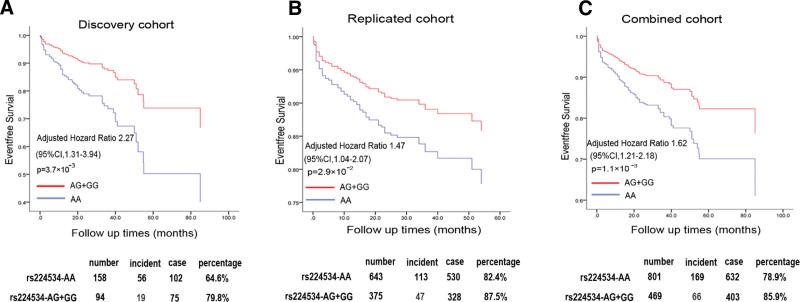
The association between genotypes of rs224534 and the outcome of HF. Cox analysis was performed to count rs224534 affect prognosis of HF in the dominant model after adjusting risk parameters (gender, age, hypertension, diabetes, hyperlipidemia, and smoking status) and beta-blocker using. Discovery (A), Replication (B), and combine cohort (C) annotate the rs224534 various genotype (red, AG + GG; blue, AA) distribution tendency.

To further evaluate the SNPs of interest associated with incident cardiovascular events of ICM in the discovery cohort, we devised a genetic screen workflow for those highlight variants. Firstly, Kaplan-Meier was used to show the distribution of genotype beside the model. According to Kaplan-Meier analysis, we choose Cox dominant proportional hazards models to further reckon adjusted for traditional risks (age, gender, hyperlipemia, hypertension, diabetes mellitus, cigarette smoking) and beta-blocker taking. 4 SNPs showed a significant association with primary events of ICM prognosis (*P* < .05). The common missense variant rs22434 from TRPV1, in the chromosome 17p13.2 region, was the top loci based on prognosis data and was significantly associated with the prognosis of ICM in Cox analysis (AG + GG VS AA, HR: 2.27, 95%CI: 1.31–3.94; *P* = 3.7 × 10^-3^) (Figs. 2B,C and 3A and Table 3). The results of Kaplan-Meier analyzed various genetic models with rs224534 and MAF in the present study were offered in Table 2, dominant is the optical model. Based on 1000 Genomes, the linkage map of rs224534 is shown in Figure 2D.

**Table 2:** The association between rs224534 and HF outcomes in discovery and replication.

Cohort	rs224534- Genotype			MAF	Additional	Dominant	Recessive
	AA	AG	GG		*P* value	*P* value	*P* value
Discovery (n = 252)	158	81	13	0.788	1.9 × 10^–2^	5.0 × 10^–3^	0.739
Replication (n = 1018)	643	317	58	0.822	0.096	3.0 × 10^–2^	0.506

**Table 3:** Results of Cox Proportional Hazard Analyses in the dominant model for Cardiac Events.

Variables		Discovery (n = 252)		Replication (n = 1028)		combined (n = 1280)	
		HR (95% CI)	*P* value	HR (95% CI)	*P* value	HR (95% CI)	*P* value
Crude	rs224534	2.05 (1.22–3.46)	6.8 × 10^-3^	1.45 (1.03–2.04)	3.2 × 10^-2^	1.60 (1.20–2.12)	1.3 × 10^-3^
Adjusted	rs224534	2.27 (1.31–3.94)	3.7 × 10^-3^	1.47 (1.04–2.07)	2.9 × 10^-2^	1.62 (1.21–2.18)	1.1 × 10^-3^
	Gender	2.25 (1.22–4.18)	9.9 × 10^-3^	0.86 (0.60–1.25)	0.441	1.11 (0.81–1.53)	0.523
	Age	1.06 (1.06–1.09)	7.0 × 10^-4^	1.04 (1.03–1.06)	9.3 × 10^-8^	1.05 (1.04–1.07)	1.3 × 10^-10^
	Hypertension	1.88 (1.13–3.13)	1.5 × 10^-2^	1.27 (0.72–2.26)	0.406	1.72 (1.24–2.39)	1.2 × 10^-3^
	Diabetes	1.43 (0.86–2.38)	0.17	1.98 (1.44–2.71)	2.2 × 10^-5^	1.78 (1.37–2.33)	1.6 × 10^-5^
	Hyperlip-idemia	1.93 (0.76–4.93)	0.17	1.45 (1.02–2.06)	3.8 × 10^-2^	0.74 (0.53–1.03)	3.3 × 10^-2^
	Current smoking	1.96 (1.12–3.41)	1.7 × 10^-2^	1.09 (0.75–1.60)	0.65	0.86 (0.63–1.17)	0.334
	β-blocker use	5.48 (2.58–11.64)	1.0 × 10^-5^	3.69 (2.63–5.17)	3.9 × 10^-14^	3.97 (2.93–5.37)	4.0 × 10^-19^

To verify the consistency of rs224534 in the discovery cohort, we performed a genotype rs224534 independent replication cohort contained 1042 ICMs. Primary incidents were overused to validate the association. We perceived significant associations of rs224534 with the prognosis of ICM. The results of Kaplan-Meier analyzed various genetic models with rs224534 and MAF in the replication study were offered in Table 2. In Cox analysis individuals carrying G allele (AG/GG genotype) had a better prognosis compared with AA homozygous (AG + GG VS AA, HR: 1.47, 95%CI: 1.04–2.07; *P* = 2.9 × 10^–2^, Fig. 3B and Table 3).

### 3.4. Combined analysis

reinvestigated this finding in a combined participator of 1280 cases was consistent with the discovery and replication population by Cox dominant hazards model. Rs224534 was significantly associated with the prognosis (AG + GG VS AA, HR: 1.62, 95%CI: 1.21–2.18; *P* = 1.1 × 10^–3^) (Fig. 3C and Table 3).

### 3.5. Expression of *TRPV1*

**Figure 4.**
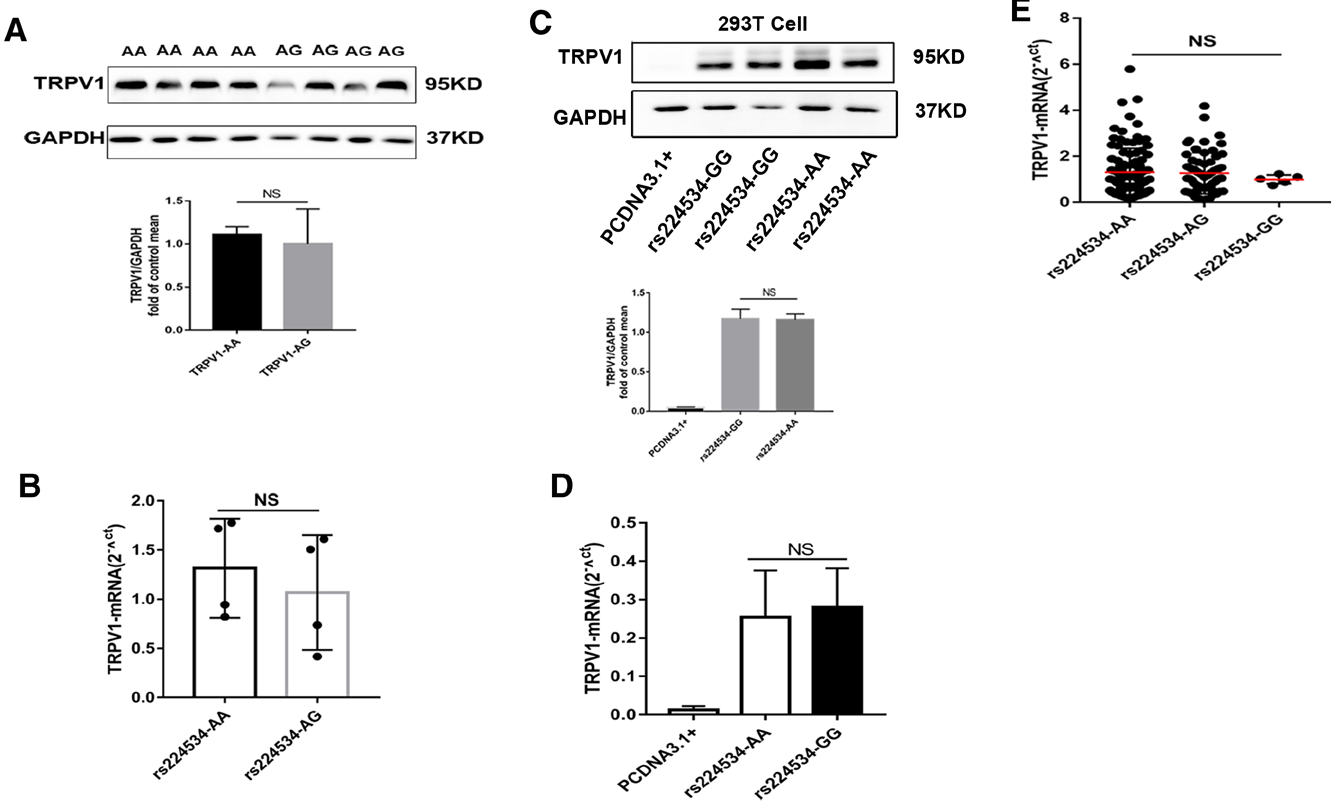
Rs224534 may not affect the expression of TRPV1. The expression level of TRPV1 on protein (A) and mRNA (B) has not differencein ICM failed human cardiac tissue with the genotype of rs224534-AA and AG. rs224534-AA (n = 4), -AG (n = 4), and the consistency of the results in 293T (C,D) which were normalized to GAPDH levels (GAPDH: glyceraldehyde 3-phosphate dehydrogenase). E. TRPV1 transcript levels in peripheral blood lymphocytes were comprised of rs224534-AA (n = 92), -AG (n = 56), and GG (n = 5) allele.

To investigate the *TRPV1* expression in the failed heart with different genotypes of rs224534 as potential functional association with ICM, we confirmed that the *TRPV1* protein in failed heart and compared the difference of rs224534 AA and AG genotype. The expression of TRPV1 had no difference in AA contrast to AG (*P* > .05, Fig. 4A,B). Subsequently, consistent results were obtained in vitro study of 293T transfection by detection of protein and mRNA level (*P* > .05, Fig. 4C,D). Meanwhile, TRPV1 mRNA levels of peripheral blood lymphocyte from 153 healthy individuals also had not existed statistical significance compared with rs224534 different allele (Fig. 4E).

## 4. Discussion

ICM is a complex and common disorder of the terminal stage of coronary heart disease (CHD) has been approved extensively that heart failure has a genetic predisposition. Currently, massive of GWAS research stick on the causal of CHD risk and common/rare gene variant that more than 60 monogenic disorder has been reported, our prior study shown functional variant associated with CHD^[20]^ and coronary artery dissection^[21]^ as well. Meanwhile, a series of genetic risk scores have made a good effort to predict the risk of individualization. While only a few studies attend to the genetic background to explore function attributed to ICM. In this study, WES was performed in ICM cases with an enlarged left ventricle (≥60 mm) and a dysfunctional heart from the current study. We performed a SKAT-O test to explore the association of TRPs and the risk of ICM. To identify the significant SNPs, Kaplan-Meier analysis and adopted COX hazards model to seek target mutations. And then, we inputted the risk factors which are convenient, noninvasive and available for clinical practice to adjust confounding factors.

The transient receptor potential cation channel subfamily V member 1 (TRPV1) located in 17p13 chromosome, as a Ca^2 +^ nonselective, permeable, cation channel is diverse distribution apart from most prevalent in sensory neurons, mediated nociception and inflammation that can be active by chemical, stress stretch, hypoosmolarity and thermal nociception.^[22]^ TRPV1 has been involved in the development of ICM,^[23]^ which plays a significant role in ischemia-reperfusion injury.^[24]^ Overall, TRPV1 played a protective role in cardiovascular diseases when it was activated.^[25–28]^ Numerous studies reported that providing cardioprotective effects for TRPV1 activated, as the dilated peptides, such as CGRP and SP, discharge from the perivascular sensory nerves innervating the myocardium by PKA/PKC pathway.^[29]^ Though, overactivation of TRPV1 may have harmful effects.^[30,31]^ There are insufficient studies on TRPV1 and the risk of cardiovascular disease, our studies showed that TRPV1 was not related to the risk of ICM.

The major findings of the current study are that rs224534, the common mission variant of TRPV1, is associated with the prognosis of ICM. In our WES study, 64 common SNPs were identified among 1868 SNVs of the TRP superfamily. With the cox dominant hazards model, rs224534-common missense mutation of TRPV1-is on the top of difference by survival analysis. Particularly, our studies found the association between rs224536 and the prognosis of ICM. Due to the false-positive, we validated it in the replication cohort to perform a multifactorial COX hazards test. After adjusting for the conventional risk factor, such as age, gender, diabetes, hypertension, hyperlipemia, smoking, and beta-blocker a classical medicine, the significant association was still existed. This result suggests that the favorable prognosis risk is rs224534-AA (mutation loci) carriers in Chinese Han. Previously work has demonstrated that the mutations of TRPV1 had been associated with Asthma,^[32]^ inflammatory disease.^[33,34]^ According to update the literature, we know that although the relationship between genetic variation and ischemic cardiomyopathy is still unclear, we have made a useful exploration.

The present study also demonstrated the impact of rs224534 on TRPV1 expression. Previous study reported TRPV1 expressed in mice cardiac muscle^[35]^ and H9C2 cell,^[36]^ however, rarely reported in humans. Specifically, our results of TRPV1 expression in human failing heart demonstrated that it had not been to the difference of TRPV1 expression with rs224534- AA and GG. Besides, previous studies were shown that rs224534 as a function of SNP in other disease.^[32]^ Sen Wang^[37]^ has demonstrated that rs224534 (G > A) was the gain function to affect the TRPV1 channel by electrophysiological assays in HEK293 cells which could be used to explain our discovered results-rs224534 AA has a better prognosis than GG. Research has reported the gain function of rs224534, but the mechanism is unclear. Our results of TRPV1 all-cDNA plasmids harboring rs224534-AA and GG loci transfected experiment verified the variant could not affect the expression TRPV1 in 239T. Therewith, we found that rs224534 (T469I) is a phosphorylation site on TRPV1 by HaploReg v4.1 and Netphos3.1 server. Hence, during sustained ischemic injury to induce activation of TRPV1, and to phosphorylate TRPV1 by T469 (rs224534) and thereby enhance its function, with consequent to enhance in cardioprotection.

### 4.1. Study limitations

There are some limitations to the current study. First, at present, the association of prognosis of ICM derived from the Chinese Han population, which needs further proved in other ethnicities. Second, in the discovery cohort, it was not distinguished between subgroups of myocardial infarction and nonmyocardial infarction while the function of TRPV1 may be different in different states of the same disease. However, they have a coincident pathophysiology process, and for the nociceptor of TRPV1 mainly mediating injury is the base function. Third, the SNPs that constructed the panel of TRP should be enriched. For example, more potential SNPs in an intron or intergenic region can be enrolled to increase candidate sizes. Fourth, our study lacks family history in traditional risk that may also be subjected to the level of medical care, and the information of disease among direct relatives has often not objective evidence or queryable medical records to support the diagnosis. Besides, the present study has not sufficiently fundamental experiments to explore the function of the TRPV1 mutation. For the moment, the emphases of research from the perspective of clinical application are to seek the association of common mutation of TRP and prognosis, and further investigations are in progress.

## 5. Conclusions

Our observations suggest that rs224534 the common missense variant of TRPV1 is associated with the prognosis of ischemic cardiomyopathy in the Chinese Han population, suggesting it may help identify newly predicted targets for ICM.

## Author contributions

Shiyang Li and Lei Xiao developed the study concept, design, and interpreted the data, and drafted the manuscript. Yang Sun performed the research; Dong Hu supervised the design of the study and revised the manuscript.

## Acknowledgments

The authors would like to thank the participators of the present study for proving understanding and support. We also thank the colleagues of the clinical follow-up office for providing a great effort.

## Supplementary Material


