# Supplementary material for: A TRPV1 common missense variant affected the prognosis of ischemic cardiomyopathy
Source: Medicine (Baltimore). 2022 Jul 29;101(30):e29892. doi: 10.1097/MD.0000000000029892 (PMC9333512; doi:10.1097/MD.0000000000029892)
Supplement: Supplementary file 1 [file medi-101-e29892-s001.pdf]

Supplementary material for

A *TRPV1* common missense variant affected the prognosis of ischemic cardiomyopathy

Methods

Recruitment for the ischemic cardiomyopathy cohort and health controls

The diagnosis of ischemic cardiomyopathy according to clinical history and coronary anatomy of patients with symptomatic HF and ICM was diagnosed based on coronary heart disease: presence of previous myocardial infarction, ≥75% stenosis in 1 or more major epicardial coronary arteries, or presence of previous percutaneous coronary intervention, coronary artery bypass grafting; left ventricular ejection fraction < 40%; left ventricular end-diameter (LVEDD) > 117% of the predicted value corrected for age and body surface area in significant coronary artery disease and a history of symptomatic HF (New York Heart Association [NYHA] functional class II or greater) between March 2003 to November 2017 (n=252). Patient data were obtained from a standardized questionnaire survey and electronic medical records according to the unified process. The exclusion criteria were as follows: non-ICM disease (defined as primary valvar heart disease, congenital heart disease, or idiopathic cardiomyopathy), severe complications such as irreversible renal or liver dysfunction, and life expectancy <1 year. Comorbidity and risk factors were: Hypertension (systolic pressure >140 mmHg, diastolic pressure >90 mmHg), Diabetes (fasting glucose >7.8mmol/L or postprandial 2 hour glucose > 11.1 mmol/L), Hyperlipidemia (total plasma cholesterol level of > 5.72 mmol/L or plasma triglyceride > 1.70 mmol/L), smoking history, taking beta-blocker >2 months. Additionally, 252 health individuals from the health examination of the community in Wuhan were enrolled in the study, with ages ranging from 50 to 88 years. The baseline demographic and family history of all study participants was obtained via standardized questionnaires. All the laboratory examinations were executed on the Rocha modular DPP system according to standard procedures at the Department of Clinical Chemistry,

Tongji Hospital.
